# Racism Counterfactuals in Health Research

**DOI:** 10.1007/s40615-025-02484-9

**Published:** 2025-06-04

**Authors:** Caryn N. Bell, Emma A. Blackson, Andrew Anderson, Kristefer Stojanovski, Bridget E. Weller

**Affiliations:** 1 Department of Social, Behavioral, and Population Sciences, Tulane University School of Public Health and Tropical Medicine, New Orleans, USA; 2 Department of Health Policy and Management, Johns Hopkins Bloomberg School of Public Health, Baltimore, USA; 3 School of Social Work, Wayne State University, Detroit, USA

**Keywords:** Racism, Counterfactuals, Health inequities, Health equity

## Abstract

There has been an increase in both research and funding aimed at exploring the connections between racism and racial health inequities. Despite these efforts, some quantitative analyses fall short when they rely heavily on counterfactual thinking (e.g., asking “what if” questions) on race and racism. Such methods are based on a theorization of race and racism that inadequately captures the White privilege inherent in the system of racism. Therefore, counterfactual thinking and methods can potentially perpetuate harmful interpretations of data. To address these shortcomings, we advocate for the use of methods that can account for White privilege including race-specific comparative analyses or intra-racial analyses. Unlike traditional counterfactual-based approaches, these types of analyses can provide a comprehensive view of how racism influences health outcomes, recognizing the varying impacts on individuals within structurally disadvantaged groups as opposed to those experienced by White individuals. Furthermore, by focusing on the specific needs of those most affected by health inequities, race-specific and intra-racial analyses adhere to principles of health equity ensuring a more accurate and effective anti-racist approach to understanding and addressing racial health inequities.

## Introduction

The poorer health outcomes experienced by Black Americans compared to White Americans have been documented for more than a century [1, 2]. There had been a dramatic increase in attention to racism and health in recent years including declarations of racism as a “public health crisis” [3, 4] and increased research funding [5]. A large literature on the detrimental effects of personally mediated or individual-level racial discrimination on health [6–10] is now discussed as part of a broader analysis of racism and health. This includes a more structural view of racism and how it creates and maintains racial health inequities [10–15]. With this marked shift toward acknowledging racism as the root cause [11] of racial health inequities [3, 4, 16], this growing health literature defines racism [17, 18], outlines how racism impacts health and health inequities [12–15, 19, 20], and discusses how to measure racism in quantitative health research [18, 21–23].

In line with the popular adage among health practitioners and scholars that “racism is the risk factor, not race” [24, 25], some researchers have adopted using the race variable in quantitative data as an indicator for racism in quantitative health analyses. For example, Lett et al. (2022) suggest that race is “a proxy for the social stratification achieved through systemic racism.” As such, the race variable may be used in quantitative analyses as a variable to measure racism. However, by implementing this approach, researchers who employ counterfactuals and treat race as a proxy for racism risk fundamentally misrepresenting the nature of racism itself.

### Race and Racism Counterfactuals

The use of counterfactuals to identify race as a causal factor of health outcomes has been debated for some time. As Glymour and Spiegelman describe in their 2017 paper on counterfactuals, “causal inferences can be drawn when the distribution of the observed outcomes among those who did not receive the intervention equals in expectation the distribution that would have been observed had those who received the intervention not received it.” Indeed, work by Rubin in 2005, specifically uses the terms “treatment” and “control” when discussing counterfactual analyses.

Previous work on race counterfactuals in the health literature has centered discussions of how race is not intrinsic or biologic as well as issues related to mediation, mutability, (non-)manipulability of race, and (non-)exchangeability of race [26–34]. Some argue that counterfactuals cannot be used to identify the causal effect of race because one’s race is not exchangeable or manipulable [31]. Others suggest this argument is based on an understanding of race rooted in biological essentialism [33].

However, in understanding racism as the cause of racial health inequities, some may assume that the previous challenges with race counterfactuals are remedied when the race variable is used as a proxy for racism. Using race as a proxy for racism, counterfactual analyses seek to use quantitative empirical methods to identify racism as a causal factor in racial health inequities. The counterfactual would determine the observed outcome among those who did receive the treatment of racism compared to those who did not receive the treatment of racism. Recent studies have sought to describe how structural racism leads to racial inequities in cardiometabolic risk as well as COVID-19 incidence and mortality by using counterfactuals where the race variable is used as a proxy for racism [26, 35]. For example, the counterfactual “What would occur if people racialized as Black were racialized as White?” understands racism as the cause of the health inequity. In these types of counterfactuals, Black Americans receive the “treatment” of racism, comparing the health outcome among Black Americans to that among White Americans who do not receive the treatment of racism. However, this understanding of racism treats the condition of being racialized as White as being outside of or unexposed to the system of racism. This fails to fully account for the pervasive influence of White privilege that fuels racial inequities in our society rooted in White supremacist beliefs and policy.

### Definitions and Understandings of Racism

Understandings of race and racism in health research has changed over time [10, 17, 36, 37]. Some definitions of racism acknowledge the role of White privilege, while others do not. Early work by Jones (2000) describes three types of racism ranging from the individual-level to the institutional-level [38]. There are also several definitions of structural racism in the health literature [13, 14, 19, 23, 39, 40]. For example, a 2017 paper by Bailey et al. defines structural racism as “the totality of ways in which societies foster [racial] discrimination, via mutually reinforcing [inequitable] systems…(e.g., in housing, education, employment, earnings, benefits, credit, media, health care, and criminal justice) that in turn reinforce discriminatory beliefs, values, and distribution of resources” reflected in history, culture, and interconnected institutions. Another well-cited paper by Gee and Ford (2011) defines structural racism as “the macrolevel systems, social forces, institutions, ideologies, and processes that interact with one another to generate and reinforce inequities among racial and ethnic groups”. Both of these definitions describe structural inequities as well as ideologies and culture that drive racial inequities.

Researchers often focus on racial inequities in a range of structural determinants as drivers of racial differences in health outcomes including racial segregation and residential environments, access to healthcare, and economic outcomes like income and wealth and how multiple structures interact to create and maintain these inequities [10–15, 19, 40–42]. Yet, researchers should not do so without considering the social processes and racist ideologies that enable these inequities. That is, as Stewart and Sewell (2011) describe, understanding racial inequities in health must include the social structures that create and perpetuate the racial inequities in health-related behaviors and resources [43].

### Considering Whiteness and White Privilege

In discussing the intellectual roots of our understanding of racism and health, Zambrana and Williams (2022) include a description of systemic racism defined by Feagin (2014) which takes a broader view of racism in the U.S. as follows: “(1) the dominant racial hierarchy, (2) comprehensive white racial framing, (3) individual and collective discrimination, (4) social reproduction of racial-material inequalities, and (5) racist institutions integral to white domination of Americans of color.” This definition of systemic racism differs from the aforementioned definitions of structural racism in that it names and centers White privilege and dominance in the system of racism in the U.S. Racism does not only oppress, marginalize, or systematically disadvantage groups that are not White. It simultaneously advantages White Americans to create and maintain White privilege and societal dominance. Jones et al. (2008) discuss the relative lack of analysis of Whiteness in health research pointing out the overall focus on the disadvantage to non-White groups caused by racism.

White privilege is not the absence of racial discrimination (i.e., the disadvantaging of Black Americans through racial discrimination), but the advantaging of White Americans above that which is just and fair. That is, Whiteness is not neutral [44]. Though some perceive White Americans as race-less making Whiteness invisible [45], White privilege can be defined as “the benefits and unfair advantage accorded to Whiteness” [46]. Additionally, Whiteness can be defined as “a cultural construction of a structural position of social advantage and power” [46, 47]. For example, studies have shown that White Americans with the lowest educational attainment have more wealth than the most educated Black Americans [48]. Moreover, studies have long shown that some birth outcomes are better for the least educated White mothers than those of the most educated Black mothers [49]. Jones et al. (2008) found that, regardless of self-identified race, those who are identified as White by others have better health outcomes. These types of studies demonstrate the White privilege inherent in Whiteness in U.S. society [44]. In addition, Kwate and Goodman (2014) examined the impacts of White privilege through the lens of Du Bois focusing on the psychosocial benefits of Whiteness that lead to health benefits. They understand White privilege to be about White people’s perception of their own status relative to others [46].

There is a long history of advantaging Whiteness socially and materially in the U.S. [45, 47]. For example, numerous historic social welfare and housing policies excluded Black Americans from benefitting from them. However, these programs led to increased economic security and wealth among the White recipients. Therefore, exclusion from these programs not only disadvantaged Black Americans but materially advantaged White Americans beyond that which would be experienced without them.

Previous studies have shown how Whiteness and White privilege (mostly) positively impacts the health outcomes of White Americans [46, 50–52]. In their work on Fundamental Cause Theory, Phelan and Link (2015) outline how advantages in “flexible resources” such as prestige or status, power, beneficial social relationships, and freedom that White Americans experience supersede the racial inequities in socioeconomic status that are often implicated in discussions of racial health inequities [11, 42, 53]. Therefore, racial inequities in determinants of health and subsequently health outcomes are not simply due to discrimination and systematic disadvantaging of Black Americans but also due to White privilege experienced by White Americans.

### Counterfactual Analyses with Race as a Proxy for Racism

In order to fully understand and develop anti-racist approaches from health research, it is critical that racism be accurately conceptualized and measured in quantitative analyses such as counterfactual thinking. Acknowledging that these systems and structures work to maintain White privilege and dominance in the U.S. is critical. Counterfactual analyses that theorize racism as a treatment that Black and other racial groups that are not White receive, by simply purporting to assess that which is lacking in Black Americans relative to White Americans, do not fully capture the ways in which racism creates and maintains racial health inequities. That is, using counterfactual-based quantitative approaches with race as a proxy for racism would suggest that White people are not affected by systemic racism at all. White Americans do not directly experience the negative disadvantages, oppression, and marginalization of living in a racist system, but White Americans directly experience the advantages of White privilege and societal dominance from living in the same racist system. Applying counterfactual terms, all Americans receive the “treatment of racism” though White Americans experience the societal advantages of Whiteness in an anti-Black racist system while Black Americans and other racial groups experience systemic marginalization, oppression, disadvantage, and structural violence. Therefore, using a counterfactual approach based in comparing outcomes among those who receive the treatment and those who do not inaccurately theorizes and understands racism.

Some might respond to this perspective by attempting to find a racial group that neither experiences the negative impacts of racism nor the benefits of White privilege. White privilege cannot be detached from Whiteness, and there are no groups that are non-White and non-Black that are race-less and outside of the system of racism. For example, there are several studies on the negative impacts of racism on the health of Native Americans and Asian Americans [54–69]. Krieger (2003) argues that the correct counterfactual for racism and racial health inequities is comparing what occurs due to racism to what would occur if racism did not exist. Asking the racism counterfactual that examines what would occur if Black people were White does not determine what would occur if racism did not exist. This counterfactual does not account for White privilege and the White supremacy that undergirds the system of racism in our society. It simply measures what Black people would experience if they were White (and had White privilege).

### Alternatives to Racism Counterfactuals in Health Research

Some might argue for analyses that seek to quantify White privilege and account for it in analyses. Others might respond by seeking to measure White privilege. As previously referenced, though few, there have been studies that empirically analyze White privilege and Whiteness among White Americans [44, 46]. However, a traditional counterfactual approach with race as a proxy for exposure to racism is not applicable here because one would not be able to account for White privilege as a confounder.

Most quantitative methods in health research stem from epidemiology, a field focused on assessing causality. Given the issues with using counterfactuals to establish race as a causal determinant that were previously described, scholars have focused on developing quantitative methods to avoid these concerns. For example, approaches like causal mediation analyses, structural equation models, joint disparity analysis, and decomposition models essentially seek to partition the race or racism effect into its constituent parts describing the pathway between the exposure of racism to racial health inequities [26, 28, 70–73]. These analyses move the understanding of race and racism away from racial essentialism toward a more social constructionist view. For example, causal mediation analyses rely on the counterfactual disparity model (i.e., the race difference in outcomes if both racial groups had the same level of the mediator) [71]. Decomposition analyses essentially compare the outcome among one group (e.g., Black Americans) if they had the same level of determinants of the outcome as the reference group (e.g., White Americans).

Instead of asking the counterfactual, “What would occur if Black people were instead White?”, causal mediation and decomposition analyses ask something like, “What would occur among Black people if their conditions (i.e., determinants of health) were the same as White people?” In comparison to traditional counterfactual thinking, we prefer using these types of analyses. Though causal mediation and decomposition analyses do not directly rely on counterfactual thinking, they do not necessarily acknowledge the role of White privilege in racial health inequities; thus, their conceptualization of racism is incomplete. That is, causal mediation and decomposition analyses that use race as a proxy for racism frame the effects of racism as racial differences in some measured or measurable determinant(s) of health. In the U.S. racism system, Black Americans having the exact same level of determinants of health as White Americans is not possible given the White privilege that inherently endows White Americans with more (e.g., those “flexible resources” described by Phelan and Link (2015) relative to other people, particularly Black Americans.

Thus, statistical analyses that acknowledge White privilege might be approaches that allow for race differences in the effects of determinants on health outcomes [74]. For example, Brown et al. (2023) examine stressors and socioeconomic status as mediators of racial differences in health. However, they use moderated mediation allowing for the pathways between socioeconomic status, stressors, and health to be moderated by racial group. Brown et al. (2023) argued that analyses of racial inequities in health that use mediation without moderation “do not take into account drastic racial differences in experiences as a result of living in a racialized society.” We agree and further suggest that this approach is a method to acknowledge the role of White privilege in racial health inequities and the effects of racism on health.

### Race-Stratified and Intra-racial Analyses

Other epistemologies and methodologies may be more appropriate for fully understanding and addressing the impacts of racism on Black health. For example, much effort may go toward empirically demonstrating that racism is causal to racial health inequities. However, with an approach that accepts racism as causal, research efforts can be used to understand how racism impacts health and identify targets for anti-racist intervention. Additionally, qualitative analyses may be more appropriate in some instances for assessing how racism creates and maintains racial health inequities. These types of shifts can ask different research questions of the problem of racism. Instead of asking “What would occur if Black people were racialized as White?”, research should be future-focused, rooted in liberation and the dismantling of racist systems rather than mired in comparing and idealizing Whiteness as the goal.

Work that quantifies structural racism as area-level racial inequities in various social determinants of health can use intra-racial analyses to examine the associations between racism and health outcomes. For example, several studies that link state- or county-level racial inequities as indicators of structural racism to various health outcomes use race-stratified analyses [75–78]. This approach acknowledges differential associations by race, thus acknowledging the role of White privilege in the impacts of racism on health and health inequities.

Intra-racial group research designs can overcome some intractable methodological problems and provide insights for interventions. It attempts to understand the impact of exposure or interventions within a specific racial group. In this case, examining a racial and/or ethnic subgroup allows the researcher to examine the impact of exposure *among* individuals with similar exposure to racism. However, it assumes that individuals have differential exposure to different levels of racism (e.g., internalized, structural, and interpersonal) based on individual differences. These individual differences can include access to resources and power (e.g., social and economic).

Previous studies of ethnicity, immigrant background, and health among Black Americans have interrogated these topics [79–83]. Studies that compare the health of US-born Black Americans to Black immigrants who come from countries that do not have a White supremacist racial hierarchy can demonstrate the impacts of racism on health. Furthermore, studies that also examine length of time living in the U.S. can additionally demonstrate the effects of living in a racist society on Black health.

Racism can also create race-specific health determinants that can impact health among Black Americans. A good example is colorism, which is discrimination experienced based on skin tone. For example, in general, Black individuals with darker complexions have poorer health outcomes than Black individuals with lighter complexions [84] Studies have also shown that health outcomes vary by skin tone among Black individuals, with evidence suggesting that people with “medium” skin tone may be the least at risk of poor health outcomes [85]. Additionally, factors like skin tone, living conditions, and socioeconomic status can be mitigating factors—albeit not always shielding an individual racialized within a particular group from the pernicious and pervasive effects of racism.

Quantitative intra-racial research designs can involve tracking the impact of an intervention or exposure on an outcome at one period and assessing changes in outcome at a later period. It can also involve comparing differences in that outcome within groups based on a particular characteristic present and varying within that racial and/or ethnic group. Within-group design can disaggregate the myriad ways racism operates and singles out a specific constitutive element (e.g., discrimination based on skin tone) that can be manipulated in an experiment (or observed to vary) within a group. May and Sen describe within-group research designs as having (1) one or more factors that are associated with racism identified as a treatment/exposure; (2) members of the group can be considered assigned to the treatment and control conditions (e.g., lives in a racially segregated neighborhood or does not); and (3) the unit of analysis is individual members of the racial or ethnic group. The advantages of the within-group approach are that it reduces the post-treatment bias problem, allows an analyst to control for confounders associated with racism, and creates better opportunities for policy intervention [86].

## Conclusions

In alignment with Monk (2022) and others [22, 43, 87], we call for “an epistemological break” [87] from a rudimentary understanding of race and racism rooted in deficit framing. Others have suggested the need for intra-racial analyses when describing the measurement of structural racism in public health research [21, 22]. In offering recommendations for measuring structural racism, Adkins-Jackson et al. (2021) describe how solely concentrating on one racial/ethnic group (i.e., intra-racial or race-stratified and comparative analyses) can operationalize how sociopolitical burdens that selected group faces contribute to inequities in health outcomes. Stewart and Sewell (2011) support the use of comparative and intra-racial analysis as one of five different methods that aim to analyze social inequalities. W.E.B. Du Bois utilized a combination of comparison-based analyses and intra-racial analyses to understand the racial health disparities in his groundbreaking work, *The Philadelphia Negro.*

Concerning theory, some implicitly reinforce the need and usage for intra-racial analysis. Born out of legal scholarship and arguably one of the most mainstream critical theories, Critical Race Theory (CRT) describes how race and racism are fundamentally entrenched within every fabric of American institutions and culture [88, 89]. One primary objective of CRT is to materialize descriptions of discrimination from racially marginalized people (i.e., people of color), or simply, centering the margins by decentering Whiteness [90–93]. Intra-racial and race-stratified analyses become imperative as it can help quantitatively ascertain descriptions of discrimination, structural racism, and White privilege. Many theories have been developed over time as a critique or expansion of CRT, some of which also speak to the need for intra-racial analysis. For example, Ford and Airhihenbuwa (2010) developed the Public Health Critical Race Praxis (PHCRP), as a semi-structured methodology of CRT for public health research and practice. One of the core phases of PHCRP is to examine contemporary racial relations, in which the research team explores how racism is operationalized in the study’s time-period [92]. A way to parse this out is to utilize race-stratified and intra-racial analysis as it would provide a meaningful way to examine how racism and White privilege have materialized in the time-period of interest.

Because race is socially constructed and varies across time and context, race and racism may not operate in the same way as in the U.S. further making the racism counterfactual insufficient in non-U.S. contexts. For example, in African contexts where applicable, the “White” racial population is a minority and has always been a racial minority, even during the colonial era. White racial minority rule set the stage for decades of continued racist and colonial practices. Examining the health impacts of global racism, anti-Blackness, and colonialism in contexts outside of the U.S. requires a shift to a relational epistemology, as scholars of colonialism and decolonization demand [94]. For example, instead of solely comparing the health outcomes of Black people to White people in African and non-U.S. contexts, scholars examine the impacts of neocolonialism and anti-Blackness on health in these contexts using qualitative and quantitative methods that do not rely only on comparing outcomes between racial groups but rather measure the manifestations or markers of racism within groups.

Intra-racial and race-stratified analyses may be limited in some contexts outside of the U.S. where race is not acknowledged or measured. Moreover, there are contexts in which race is not a societally salient construct. There are also limitations to the use of intra-racial analyses specifically when a measure of a type of racism does not exist or does not vary greatly within a racial group.

As demonstrated by these limitations, though race-stratified and intra-racial analyses sound relatively straightforward, the explanations of why it is needed described here belie a complexity that requires inter- or trans-disciplinary knowledge and commitment to anti-racism both in thought and in deed. The problem of “health equity tourism” has been highlighted recently [95]. Because systemic racism is a seemingly perpetual organizing system of the U.S. and because systemic racism changes over time, it is critical to continue to conduct analyses that compare outcomes among Black Americans to White Americans because they demonstrate the existence of unjust and unfair systemic racism in the U.S. However, advancement toward eliminating racial health inequities will remain at the level of discussion and debate without the correct framing, understanding, and analyses of the impacts of racism on racial health inequities.

Counterfactual analyses must correctly understand and evaluate racism. It is imperative that scholars who seek to understand the impacts of racism on racial health inequities are committed to accurately understanding racism in the U.S. and its role in racial health inequities toward achieving health equity and health justice. We discourage the use of counterfactual thinking to determine the effects of racism on health and racial health inequities because they do not acknowledge the role of White privilege inherent in the system of racism. That is, traditional counterfactual methods understand racism as a “treatment” that Black and other non-White racial groups receive allowing for White Americans to be the “control” group. This is an inaccurate understanding of racism in the U.S. To acknowledge the role of Whiteness in racial health inequity, we recommend the use of quantitative methods that acknowledge and examine the potential for racial differences in the impacts of health determinants such as moderated mediation analyses. We also suggest the use of race-stratified and intra-racial analyses as well as non-quantitative methods to assess the impact of racism on health and racial health inequities.
